# Segmentation of hepatic vessels from MRI images for planning of electroporation-based treatments in the liver

**DOI:** 10.2478/raon-2014-0022

**Published:** 2014-07-10

**Authors:** Marija Marcan, Denis Pavliha, Maja Marolt Music, Igor Fuckan, Ratko Magjarevic, Damijan Miklavcic

**Affiliations:** 1 University of Ljubljana, Faculty of Electrical Engineering, Ljubljana, Slovenia; 2 Institute of Oncology Ljubljana, Ljubljana, Slovenia; 3 Clinical Department for Diagnostic and Interventional Radiology, Clinical Hospital “Dubrava”, Zagreb, Croatia; 4 University of Zagreb, Faculty of Electrical Engineering and Computing, Zagreb, Croatia

**Keywords:** electrochemotherapy, non-thermal irreversible electroporation, treatment planning, hepatic vessel segmentation, non-invasive tumor treatments, MRI of liver

## Abstract

**Introduction.:**

Electroporation-based treatments rely on increasing the permeability of the cell membrane by high voltage electric pulses delivered to tissue via electrodes. To ensure that the whole tumor is covered by the sufficiently high electric field, accurate numerical models are built based on individual patient geometry. For the purpose of reconstruction of hepatic vessels from MRI images we searched for an optimal segmentation method that would meet the following initial criteria: identify major hepatic vessels, be robust and work with minimal user input.

**Materials and methods.:**

We tested the approaches based on vessel enhancement filtering, thresholding, and their combination in local thresholding. The methods were evaluated on a phantom and clinical data.

**Results:**

Results show that thresholding based on variance minimization provides less error than the one based on entropy maximization. Best results were achieved by performing local thresholding of the original de-biased image in the regions of interest which were determined through previous vessel-enhancement filtering. In evaluation on clinical cases the proposed method scored in average *sensitivity* of 93.68%, *average symmetric surface distance* of 0.89 mm and *Hausdorff distance* of 4.04 mm.

**Conclusions:**

The proposed method to segment hepatic vessels from MRI images based on local thresholding meets all the initial criteria set at the beginning of the study and necessary to be used in treatment planning of electroporation-based treatments: it identifies the major vessels, provides results with consistent accuracy and works completely automatically. Whether the achieved accuracy is acceptable or not for treatment planning models remains to be verified through numerical modeling of effects of the segmentation error on the distribution of the electric field.

## Introduction

Exposing a biological cell to a sufficiently high electric field causes increased permeability of the cell membrane. This increased permeability of the membrane allows transfer of molecules which normally lack membrane transport mechanism into the cell. The described effect of the electric field on the cell is called *electroporation.* 1,2 Electroporation can be classified as either reversible or irreversible. The reversible/irreversible nature of electroporation is in strong correlation with pulse amplitude, duration and number of pulses. In reversible electroporation, the cell membrane eventually returns to its normal state. Irreversible electroporation however leads to cell death because the cell membrane is permanently disrupted or due to the extensive loss of the intracellular components. Combination of reversible electroporation with traditional methods of chemotherapy has resulted in a new method for tumor treatment named *electro-chemotherapy* (ECT).^3^–^5^ Irreversible electroporation (IRE) has found its application in tumor treatment as a tissue ablation procedure, its main advantage being the fact that, if controlled properly, it does not thermally damage the tissue.^6^–^8^

Tumor treatments based on electroporation like ECT and IRE include placement of the electrodes in the tissue and delivery of the electric pulses. In order for the treatment to be successful the whole tumor must be covered by a sufficiently high electric field. The magnitude and distribution of the electric field depends on the number and the position of the electrodes, the amplitudes of pulses applied per electrode pair and the electric properties of the tissue, especially conductivity.^9^,^10^

Prediction of parameters needed for successful treatment is easier for surface tumors which is why the ECT was first performed on skin tumors.^4^ Ensuring the complete tumor coverage with a sufficiently high electric field is however more challenging in the case of deep-seated solid tumors as well as large tumors.^11^ This was well demonstrated in a case where a patient with a deep-seated tumor in the thigh was treated with ECT.^12^ The post-treatment evaluation showed that 6% of the tumor volume was not covered by a sufficiently high electric field, which caused the tumor to re-grow. The reasons which reduce predictability of the electric field distribution in deep-seated tumors are the tumor position, high diversity in tumor size and shape, and presence of the surrounding tissues with different electric conductivities. Predictability of an adequate distribution of the electric field can be best achieved by calculating a patient-specific treatment plan as a part of an electroporation-based treatment procedure.^13^ A patient-specific treatment plan for electroporation-based treatment of deep-seated solid tumors takes into account patient geometry and tissue properties to generate an optimal set of treatment parameters.^14^,^15^

Correctness of a treatment plan is ensured by an accurate model of the patient which includes the tumor with critical surrounding tissues and structures. The patient model is built by segmenting the medical images and then used to perform numerical calculations of the electric field distribution. A proof-of-concept was provided in a clinical study in which colorectal metastases in the liver were treated by means of ECT.^16^ For the purpose of the mentioned clinical study, an algorithm for automatic segmentation of the liver from MRI images was developed.^17^ Similar treatment planning process is well-established in radiotherapy where it has been in use for decades.^18^ Generation of models from medical images for subsequent numerical calculations has also been used as a part of treatment planning for radiofrequency ablation (RFA) of liver tumors.^19^,^20^

Other than liver and tumor tissue, critical structures that need to be included in the model for both RFA and electroporation-based treatments of the liver are hepatic vessels. For the purpose of radiofrequency ablation, vessels which measure more than 3 mm in diameter size have been described as critical because of their influence on heat propagation.^21^ In case of electroporation-based treatment of the liver the hepatic vessels are important for other reasons. Firstly, the electric conductivity of the vessels is different than that of the liver tissue and tumors, which can have an impact on the electric field distribution, especially in cases when a tumor is situated close to large vessels.^22^ Secondly, during an electroporation-based treatment the surgeons insert needle electrodes into the liver tissue and these should not damage larger hepatic vessels. The hepatic vessels which were identified by surgeons as critical are vena cava and vena porta with branches up to second order, left, middle and right hepatic vein, and larger hepatic arteries. These vessels will thus be the ones we will most certainly want to include in our model. Lastly, the model of vessels built from medical images can be used for intra-operative visualization to help surgeons navigate during the insertion of the electrodes.

The problem of segmentation of vessels in general^23^ and hepatic vessels in particular has been an area of interest for several decades. The interest in segmentation of hepatic vessels resulted in exploring several different approaches. First attempts were based solely on thresholding^24^ and region growing.^25^,^26^ The evolution of highly popular methods for enhancement of tubular structures^27^–^29^ resulted in their combinations with thresholding^30^ and region growing.^20^,^31^–^33^ Other than tube-enhancing filtering the traditional methods of segmentation were enhanced through use of Gaussian mixture models^34^,^35^ and by utilizing morphology of the vascular tree through centerline extraction.^20^,^31^ More advanced methods for segmentation of hepatic vessels include those based on graph-cuts^36^,^37^, active contours^38^ and morphological properties embedded in context-based voting system.^39^

All these methods for vessel segmentation have however been designed for and applied to CT images. To our knowledge, no method so far was tested on MRI images. Although CT images have been considered superior for hepatic vessels, the vessels are also visible in MRI images, especially when a contrast agent is applied. With respect to the colorectal metastases of the liver, multiple studies have shown that MRI is superior to CT in sensitivity and accuracy of detecting tumor lesions.^40^–^45^ If MRI is a modality of choice for detecting the tumors, using the same modality to segment the hepatic vessels would avoid the need for registration and errors that inevitably come with it. Another reason why MRI is a method of choice for planning of electroporation-based treatments is possibility to directly observe the distribution of the electric field using the magnetic resonance electric impedance tomography (MREIT), which was described in the work of Kranjc *et al*.^46^,^47^ and is being actively explored.

Given all of the mentioned advantages of MRI over CT in treatment of colorectal metastases in the liver with electroporation-based treatments, we directed our research towards segmentation and validation of segmentation of hepatic vessels from MRI images. The segmentation method used for hepatic vessels has to be robust and include minimal or no user interaction. These prerequisites are necessary for using the procedure for hepatic vessel segmentation as a module in the process of treatment planning.^48^ Having this in mind, the segmentation methods we tested were built upon already established and robust approaches based on filtering, vessel enhancement, automatic thresholding and region growing. Data used in validation consisted of two sources: a phantom and clinical cases. The phantom was used to optimize the segmentation parameters and analyze the performance of methods in detail. Images of clinical cases were then used to validate the performance of segmentation methods under realistic conditions.

## Materials and methods

### Segmentation of hepatic vessels

In order to segment the hepatic vessels from MRI images we tested several simple approaches, alone and their combinations. The main approaches include vessel enhancement filtering, thresholding, region growing, connected component analysis and morphological operations.

To determine the optimal method for our purpose we tested two different thresholding methods on different input: on original de-biased images and on the results of vessel enhancement filtering. The thresholding of that input was performed on the slice level and is referred to as *global* thresholding. The thresholding method that performed best on phantoms was also tested *locally* on smaller regions of original de-biased images determined by vessel enhancement filtering.

#### Pre-processing phase

Prior to running any of the methods on the original images we performed de-biasing in order to remove the inhomogeneity of image intensity. The intensity inhomogeneity is a product of the magnetic field inhomogeneity in the MRI device.^49^ The applied de-biasing method is publicly available and based on the work of Zheng *et al*.^50^ After de-biasing the images were masked with the results of liver segmentation.^17^

#### Vessel enhancement filtering

The filter we used is based on the work of Frangi *et al*.^28^ The filter differentiates line-like from blob-like and plate-like structures by observing the relationships between eigenvalues of the Hessian matrix in each voxel of the image. Before applying the filter, the image is scaled by filtering with Gaussian kernels of different size σ. The value of σ is set to a value that equals the size of the diameter of the vessels we wish to enhance.

For each scale σ the probability of a voxel belonging to a line, i.e. *vesselness* is calculated as:

(1) $$\begin{array}{lll} & \mathrm{if} & \lambda_{2}>0\|\lambda_{3}>0\\ \nu (\sigma )=\{\begin{array}{c} 0\\ \left(1-\exp\left(-\frac{R_{a}{}^{2}}{2\alpha^{2}}\right)\right)\exp\left(-\frac{R_{b}{}^{2}}{2\beta^{2}}\right)\left(1-\exp\left(-\frac{S^{2}}{c}\right)\right) \end{array} & \mathrm{else} & \end{array}$$

where |λ_1_|≤|λ_2_|≤|λ_3_| are eigenvalues of the Hessian matrix in three dimensions, $R_{a}=\frac{\left|\lambda_{2}\right|}{\left|\lambda_{3}\right|}$ and $R_{b}=\frac{\left|\lambda_{1}\right|}{\sqrt{\left|\lambda_{2}\lambda_{3}\right|}}$ are parameters which discriminate line-like structures from plate-like and blob-like structures, and $S={\left\|H_{\sigma }\right\|}_{F}=\sqrt{\sum_{j}{\lambda_{j}}^{2}}$ is the Frobenius norm of the Hessian matrix. Values of parameters α and β were chosen through optimization on phantoms and were selected as 0.3 and 0.7, respectively. Value of parameter c is calculated for each case and for each value of scale σ according to the following equation:

(2) $$c=\frac{1}{2}{\left\|H_{\sigma }\right\|}_{F}=\frac{1}{2}\sqrt{\sum_{j}{\lambda_{j}}^{2}}$$

The parameter c is used in the expression for vesselness as is, without squaring and multiplying by 2 as it is done in the original work of Frangi *et al*.^28^ The reason for this is better enhancement of vessel structures. The final vesselness filtered image is obtained by calculating the maximum of vesselness values at different scales for each voxel of the image.

#### Thresholding

Out of many thresholding methods developed until today we chose to implement two of the most successful as reported by Sankur *et al*.^51^ The first method is based on minimizing intra-class variance.^52^ The second method is based on maximization of image entropy.^53^ Both methods are completely automatic and were implemented to determine the threshold on slice level on an image histogram with values in the 16-bit range.

We assessed the performance of the two thresholding methods globally on de-biased original images and vesselness filtered images of both phantoms and clinical cases. Additionally we assessed the method that performed better globally on smaller regions of interest. The details of local thresholding are described in the section *Proposed method*.

#### Proposed method

Through analysis of the results of previously described methods applied on both phantom and clinical data, we derived a method comprised of the best aspects of vessel enhancement filtering and thresholding. Vessel enhancement filtering is excellent for locating the position of the vessels but unable to determine their exact borders. Thresholding of the de-biased original image can detect vessel borders but not with consistent accuracy throughout the whole image. The proposed method is therefore based on *local* thresholding of smaller regions of interest (ROI), rather than deriving a single threshold for the whole slice. The ROIs for local thresholding are determined based on the output of vessel enhancement filtering. Detailed steps of the proposed method with all the input, output, parameters and dimension in which the step is performed are provided in Table 1.

First, we performed sinc interpolation to obtain isotropic voxels (O2) so that we could perform vesselness filtering.^54^ After that we applied the vesselness filtering on the interpolated, de-biased, masked original images (O3). The filtered image (O4) was interpolated once again to obtain the original voxel size (O5). In the end of the filtering section the result of vesselness filtering has to be once again masked with the original liver mask to suppress the high response which appears in the area where background borders with the liver (O6).

The output of the vesselness filtering has high values for voxels with high probability of belonging to a vessel and is very low for very small vessels. The voxels with small vesselness values might also be a result of image noise. For this reason we have chosen a small threshold of 0.05 of the maximum vesselness value. All of the voxels with a vesselness value higher than this threshold are isolated into a basic vessel model (O7). A basic vessel model thus consists of voxels with high certainty of belonging to a vessel. The same small threshold for output of Frangi’s vessel enhancing filter was already successfully used in the work of Dongen *et al*. to prevent false positives in the algorithm for extraction of pulmonary vasculature.^55^

To eliminate the smallest vessels which are not of interest for electroporation-based treatments we morphologically open the results to remove all objects with a diameter smaller than 3 mm. We need to keep only larger objects (O8) because the smallest hepatic vessels from the list of those that should not be damaged during the electrode insertion are the main hepatic arteries, and they measure around 3 mm in diameter and more.^56^

After we have extracted and morphologically opened the basic vessel model (O8) we proceed with local thresholding to determine the exact vessel borders. To extract the ROIs we first perform the connected component analysis on the slice level to break the basic vessel model into smaller 2D objects (O9). These objects are then morphologically dilated with a structuring element in the shape of a disc with a radius of 5 pixel. The dilation gives us the ROIs (O10) within which we perform the local thresholding (O12). The threshold for each ROI is calculated based on variance minimization.

The final steps in the proposed method are meant to refine the results by adding possibly missed nearby voxels and removing small objects. For this purpose we perform region growing with results of local thresholding (O12) as seed points. Region growing is performed on de-biased original images in 3D by searching the 27-neighborhood of each seed voxel. A threshold for adding new voxels is determined on a slice level as a median value of intensities of voxels already marked as vessels. The thresholds are re-calculated after each series of newly found voxels. The region growing stops once there are no new voxels that could be added.

After region growing we mask the results (O13) with an eroded liver mask (O14) to eliminate boundary outliers. This step is in general unnecessary if the provided liver masks are completely accurate and contain only liver voxels. Otherwise the results of the vessel segmentation will also include a small strip around the liver which in original images is of similar intensity as vessels. The final step in the proposed method includes once again morphologically opening the results (O15) to remove all objects with a diameter smaller than 3 mm.

**FIGURE 1.**
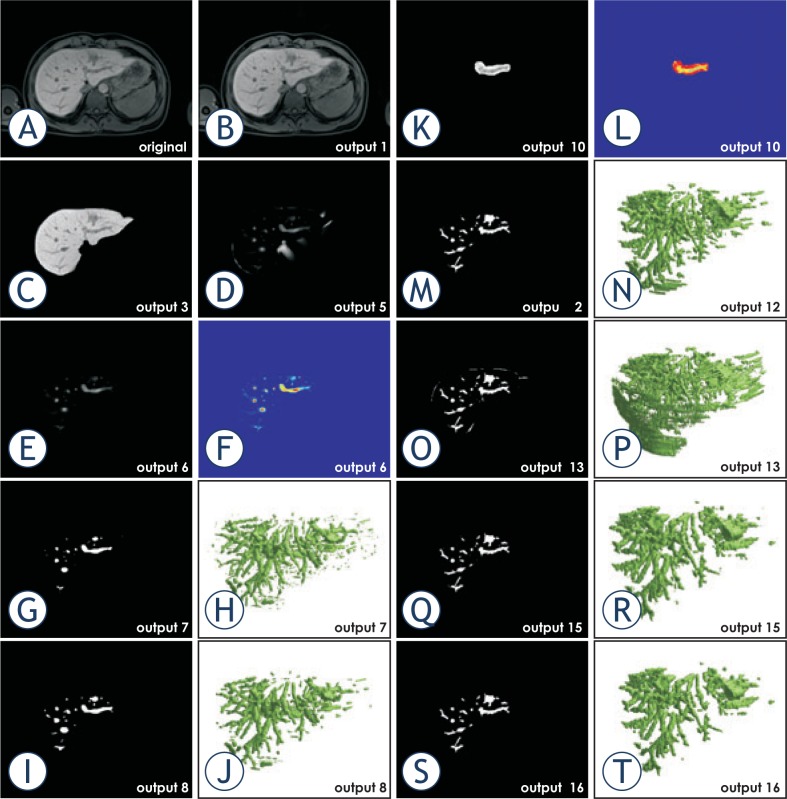
Output of the proposed method applied on a clinical case. **(A)** Original image. **(B)** De-biased original image. **(C)** Masked de-biased original image. **(D)** Vesselness filtered image. **(E)** Masked vesselness filtered image. **(F)** The same output as in E. presented in colored scale. **(G)** Basic vessel model with small objects. **(H)** Basic vessel model with small objects shown in 3D. **(I)** Basic vessel model without small objects. **(J)** Basic vessel model without small objects shown in 3D. **(K)** Basic object with ROI. **(L)** Basic object with ROI in colored scale. **(M)** Result of local thresholding. **(N)** Result of local thresholding shown in 3D. **(O)** Result of region growing. **(P)** Result of region growing shown in 3D. **(Q)** Result of masking with an eroded mask. **(R)** Result of masking with an eroded mask shown in 3D. **(S)** Final result after the removal of small objects. **(T)** Final result after the removal of small objects shown in 3D.

In order to give better insight into the proposed method we provide output of all the relevant steps in Figure 1. The outputs are the result of applying the proposed method on a clinical case. For all of the presented steps we provide a one slice output and for some steps also the complete 3D model, if relevant.

Most of the parameters that are used in the proposed method are calculated automatically based on the image. These parameters include the two most critical parameters: the value of c used in the vessel enhancing filter that influences the filter output most^57^ and thresholds in the local thresholding step. Values of two of the remaining parameters of the vessel enhancing filter, α and β, were chosen based on validation on phantoms. The rest of the parameters that had to be set do not directly influence the accuracy of the results but rather determine the region of interest in which the main parameters operate. These less-sensitive parameters are namely a threshold in step 7 and a structuring element in step 9 (Table 1). We would, however, not suggest setting these parameters to more than ±25% of the values used in this paper. There is one parameter remaining that strongly influences the output of the vessel enhancement filter, and that is the value of σ in the Gaussian kernel. This parameter needs to be set only once and according to the size of the vessels one wishes to extract, as is stated in the work of Frangi *et al*.^28^

#### Phantom design

**FIGURE 2.**
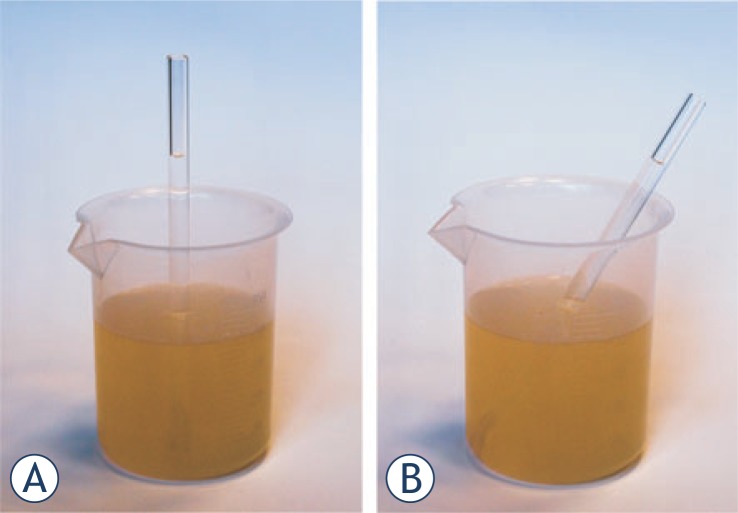
A simple phantom constructed for validation of hepatic vessel segmentation from MRI images. The phantom is made of agarose gel and a glass tube filled with physiological solution inserted in: **(A)** perpendicular position. **(B)** tilted position.

Our primary concern in hepatic vessel segmentation was the accuracy of segmentation rather than the segmentation sensitivity to the depth of the vessel tree. For this reason we created a phantom which enabled detailed observation of whether a certain method over- or undersegments. The phantom was composed of a cup filled with agarose gel and a tube filled with physiological solution inserted into that gel, similar to the work of Merkx *et al*.^58^ and Jiang *et al*.^59^ The gel was prepared as a 0.5% solution of agarose in 100 ml distilled water, doped with 0.17 mM MnCl2 to enhance MRI signal properties.^60^ Three glass tubes with outer diameters of 4, 6 and 8 mm were filled with physiological solution in order to model the vessels. Each tube was inserted into its own cup filled with agarose gel perpendicularly to the cup bottom. Another set of tubes, also with 4, 6 and 8 mm outer diameters were inserted into another three cups filled with agarose gel, but this time in a tilted position. In total, we had six cups containing tubes. An example demonstrating different positions of the tube inside the cup is shown in Figure 2. All six cups were placed in a Siemens Avanto 1.5T MRI device and imaged at the same time in order to ensure the same imaging conditions for all six phantoms. Imaging parameters were set as in the standard protocol for imaging of the abdomen: T1-weighted, VIBE breath-hold, coronal plane with body coil. We imaged in two intra-slice resolutions: 1.56 mm by 1.56 mm and 1.04 mm by 1.04 mm. The slice thickness in both cases was 2 mm.

In order to be able to directly compare different vessel diameters imaged with different resolutions, we expressed the vessel diameters in *number of pixel/diameter* instead of in mm. The same approach was already used in papers which also evaluated accuracy of determining vessel area from MR angiography data.^58^,^59^ When expressed in pixel/diameter, the value of different resolutions we observed were 2.56 pix, 3.84 pix, 5.12 pix, 5.76 pix and 7.69 pix per diameter.

#### Clinical data

For validation on clinical data we obtained MRI images of six patients that were a part of Phase I/II clinical study “Treatment of Liver Metastases with Electrochemotherapy (ECTJ)” (EudraCT number 2008-008290-54; ClinicalTrials.gov (NCT01264952)).^16^ The study was conducted at the Institute of Oncology Ljubljana, Ljubljana, Slovenia. Regulatory approvals from the Institutional board, as well as from the National Medical Ethics Committee were obtained. Written consents of the patients were obtained. The series on which we performed the segmentation were the ones on which the colorectal metastases are most visible. The segmentation of the liver was also performed on the same image series by a method described by Pavliha *et al*.^17^ The reason for choosing the series where the liver, hepatic vessels and colorectal metastases are all visible was to avoid the need for subsequent registration. The chosen series were T1-weighted, VIBE breath-hold, transversal plane, with body coil and imaged 20 minutes after injection of the Primovist® (Bayer Group, Germany) contrast agent. Images were acquired with a Siemens AVANTO 1.5T MRI device at the Institute of Oncology in Ljubljana. In three cases the inter-slice resolution was 0.684 mm by 0.684 mm with a slice thickness of 2.75 mm. In the other three cases the inter-slice resolution was 1.188 mm by 1.188 mm with a slice thickness of 3 mm.

### Accuracy assessment metrics

#### Phantom data

**FIGURE 3.**
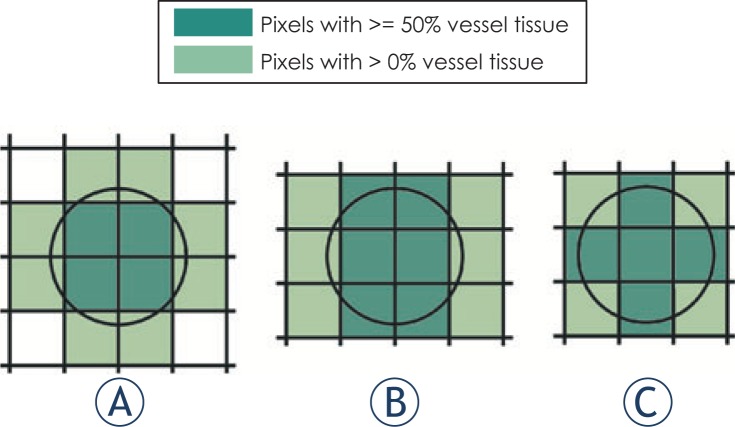
Theoretical model of reference vessel area with all possible positions of the object relative to the sampling grid. An example for the 2.56 pixel/diameter resolution. **(A)** vessel with a center in the pixel point, **(B)** vessel with the center on the middle of one of the pixels’ edges, **(C)** vessel with a center position right in the middle of one of the pixels. The pixels with >= 50% vessel tissue are a subset of pixels with >0% vessel tissue.

Once the images have been segmented, we counted the number of pixel characterized as ‘vessel’ in each slice, thus obtaining the *segmented vessel area*. For *reference vessel area* we created a theoretical model which observes different ways in which a perfect circle can be positioned relative to the sampling grid of certain size, depending on the circle size and the grid size. The illustration of our theoretical model for the case of 2.56 pixel/diameter is presented in Figure 3.

The need for such theoretical model is caused by *partial volume effect*, i.e. an artifact in medical imaging where the value of a border pixel between different tissue types is influenced by the amount of tissues it is composed of. After segmentation, the pixel can be characterized as belonging to only one tissue type. Which type will it be depends on the amount in which a certain type is present in the pixel, but also on the segmentation method. Some segmentation methods, for instance, would characterize every pixel that contains any amount of vessel as a vessel pixel.^59^ We have therefore defined three reference area values in our theoretical considerations. The first value, the *optimal vessel area* is the number of all pixels which contain at least half of the vessel tissue (pixels marked darker in Figure 3). The second reference value, the *maximum vessel area* is the number of all pixels that contain any amount of the vessel tissue (all colored pixels in Figure 3). The third reference value is *calculated vessel area* which is the mathematically calculated area of the perfect circle, expressed in number of pixels. The three reference area values (optimal, maximum and calculated) are calculated for each resolution and each of the three positions of object illustrated in Figure 3. The final *optimal vessel area* for a certain resolution is the smallest of the optimal vessel area calculated for three positions (in Figure 3 that would be 4 pixels in case A).The final *maximum vessel area* for a certain resolution is the largest of the maximum vessel area calculated for three positions (in Figure 3 that would be 12 pixels in cases A and B).

If the number of pixel contained in segmented vessel area falls in the range between *optimal vessel area* and *maximum vessel area*, we consider the segmentation valid. Otherwise, we count the number of pixels outside this range as *pixels missed*. The segmentation error is expressed as *relative area error*, in percent:

(3) $$\text{relative}\;\text{area}\;\text{error}\;[\%]=\frac{\text{number}\;\text{of}\;\text{pixels}\;\text{missed}}{\text{calculated}\;\text{vessel}\;\text{area}}*100\%$$

#### Clinical data

The gold standard for the evaluation of segmentation of clinical data is a segmentation performed by manually determining an optimal threshold for each slice and manually drawing possibly missed contours. The segmentation result was additionally validated by one of the authors (MMM) who is an experienced radiologist and who manually adjusted the segmentation where necessary. The evaluation of the segmentation of clinical data was performed on the level of objects detected in individual slices.

Metrics used for validation included a hit rate, i.e. a ratio of the number of detected objects against the number of all objects in the image, sensitivity (SEN = true positives / (true positives + false negatives)), average symmetric surface distance (ASSD)^34^,^61^ and Hausdorff distance. ASSD provides a measure of the average mutual distance between edges of the two surfaces, while Hausdorff distance is in fact the maximum symmetric surface distance. ASSD and Hausdorff distance were calculated according to equations (4) and (5), respectively:

(4) $$\mathrm{ASSD}=\frac{\sum_{a\in A}\underset{b\in B}{\min}\left\|a-b\right\|+\sum_{b\in B}\underset{a\in A}{\min}\left\|a-b\right\|}{N_{A}+N_{B}}$$

(5) $$H(A,B)=\max\left\{\underset{a\in A}{\max}\left(\underset{b\in B}{\min}\left\|a-b\right\|\right),\underset{b\in B}{\max}\left(\underset{a\in A}{\min}\left\|a-b\right\|\right)\right\}$$

where A and B denote the borders of segmented and reference objects, a and b are points on A and B respectively. ‖*a*−*b*‖ denotes the distance between a and b. N_A_ and N_B_ are the number of points on A and B.

We have chosen to use ASSD and Hausdorff distance to describe the segmentation specificity rather than a measure of specificity itself (SPEC=true negatives / (false positives + true negatives)) since a high number of true negatives (background) would always yield a high value of specificity. The values of sensitivity, ASSD and Hausdorff distance for the whole image were obtained by calculating a median of those values for all objects. We calculated the median instead of the mean since the data did not conform to Normal distribution.

Additional to previously described metrics we also used the receiver operating characteristics (ROC) curve analysis to objectively compare results of image filtering with original and de-biased images.^62^–^64^ The ROC curves used were constructed with threshold as a parameter.

## Results

The first part of this section shows the results of segmenting phantom images. There we assess the performance of thresholding algorithms based on variance minimization and entropy maximization on original and vesselness filtered images. The second part shows the results of segmenting images obtained from clinical cases. In this part we present the comparison of methods that performed best on phantoms and the method based on local thresholding, i.e. the proposed method.

### Images of phantoms

**FIGURE 4.**
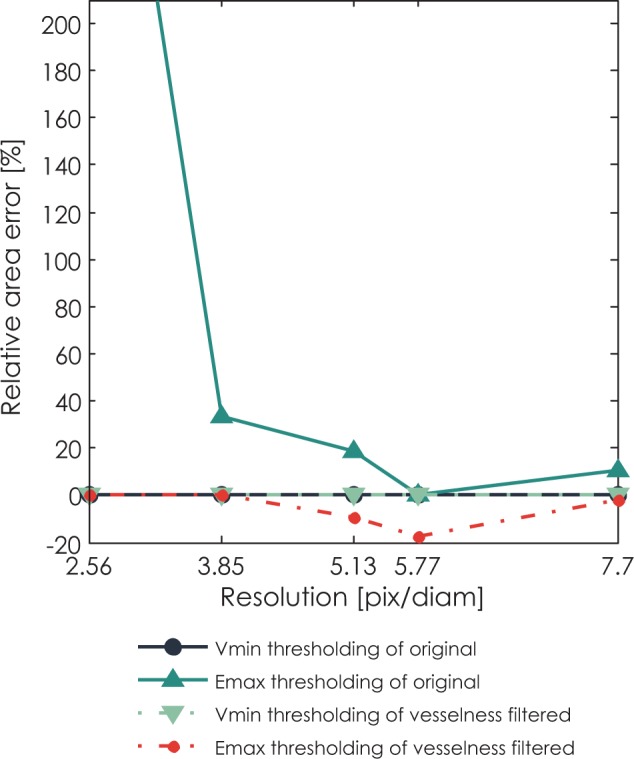
Median accuracy of segmented area of phantom in perpendicular position as a function of resolution for different segmentation methods: variance minimization thresholding of the original image, entropy maximization thresholding of the original image, vesselness filtered image thresholded by variance minimization thresholding, and vesselness filtered image thresholded by entropy maximization thresholding.

For the validation on phantom data, Figure 4 shows relative area error of different segmentation methods for segmentation of tube phantom in perpendicular position under different image resolutions. The thresholding method based on variance minimization produces 0% error on both original images and vesselness filtered images. The thresholding method based on entropy maximization undersegments the vesselness filtered images and highly oversegments the original images. As could be expected, an absolute value of error drops with increasing resolution.

**FIGURE 5.**
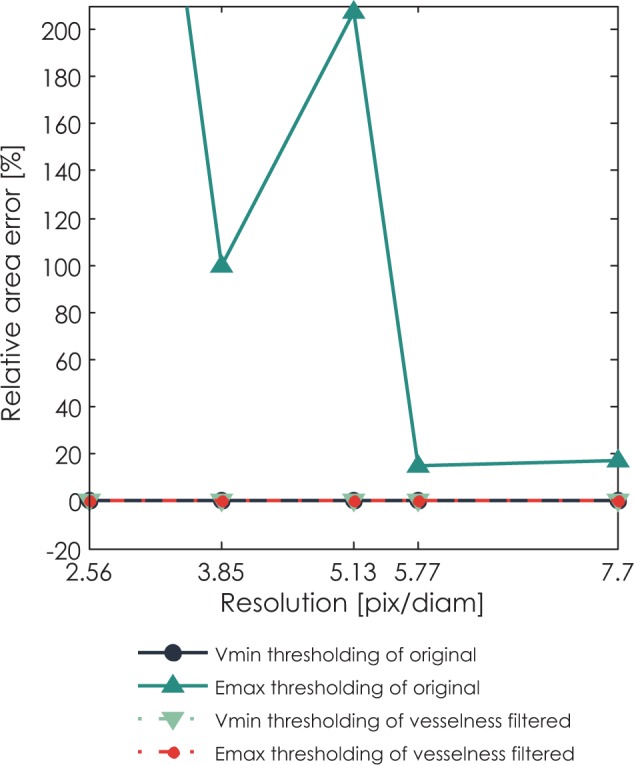
Median accuracy of segmented area of phantom in tilted position as a function of resolution for different segmentation methods: variance minimization thresholding of the original image, entropy maximization thresholding of the original image, vesselness filtered image thresholded by variance minimization thresholding, and vesselness filtered image thresholded by entropy maximization thresholding.

Figure 5 also shows the same error as Figure 4, only for the phantom in tilted position. The thresholding method based on variance minimization again produces 0% error on both original images and vesselness filtered images. The thresholding method based on entropy maximization produces an error only in the case of original images, which as expected drops with increasing resolution. Notably, an absolute error of the method based on entropy maximization applied on original images is higher for the phantom with tube in tilted position than for the phantom in perpendicular position. An error for the same thresholding method applied on vesselness filtered images in the case of tilted phantom is 0%.

In conclusion, thresholding based on variance minimization outperformed the thresholding based on entropy maximization on both original and vesselness filtered images.

### Images of clinical cases

In this section the thresholding method that performed best on images of phantoms, which is thresholding based on variance minimization, was also applied to images of clinical cases. The thresholding was first applied globally on de-biased original images and on vesselness filtered images. After that we applied our proposed method which is based on local thresholding.

**FIGURE 6.**
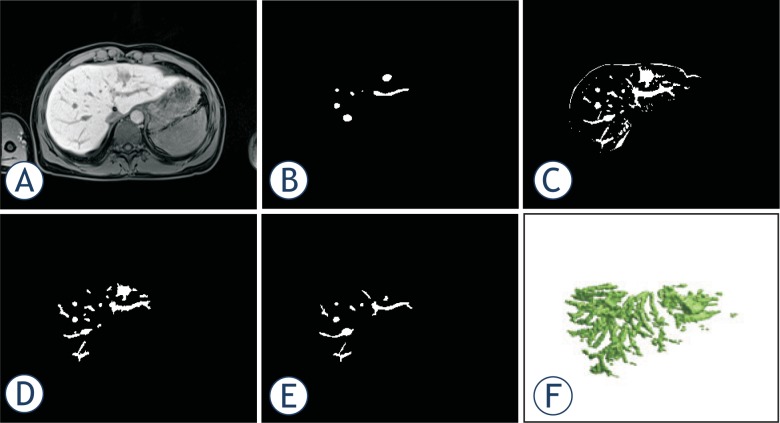
Visual comparison of performance of global thresholding and our proposed method. **(A)** Original image slice. **(B)** Results of variance minimization based global thresholding of the vesselnes filtered image. **(C)** Results of variance minimization based global thresholding of the de-biased original image **D**. Results of our proposed method. **(E)** Gold standard – a radiologist segmentation. **(F)** 3D result of the segmentation by the method in **(D).**

Based on visual inspection only it was possible to determine that direct global slice-by-slice thresholding of vesselness filtered images results in large undersegmentation of the vessels. The segmentation inaccuracy is shown in Figure 6, where Figure A shows one slice of the original data while Figure B shows the result of the segmentation of the same slice using thresholding of the result of the vesselness filter. Figure 6.C shows the result of global thresholding of the de-biased original slice based on minimization of variance. Although more accurate than thresholding of the vesselness filtered image, this approach detects many false positives on the liver border. In Figure 6.D we can observe that false positives from Figure 6.C can be avoided by our proposed method which also provides the highest level of accuracy. Figure 6.E shows the gold standard – the radiologist segmentation. Figure 6.F is a 3D representation of the segmentation by the proposed method.

**FIGURE 7.**
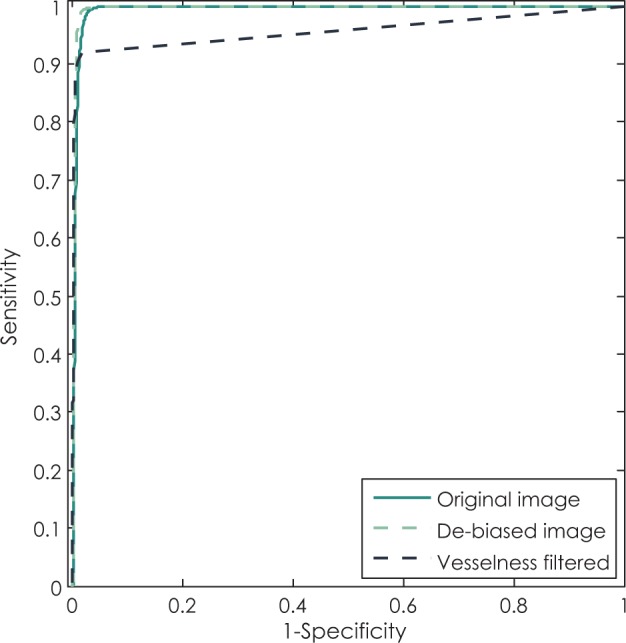
Demonstration of performance of simple binary classifier (thresholding) on original image, original image with removed bias and vesselness filtered image using ROC curves.

To additionally explore the potential of differently filtered images at providing an accurate segmentation, we observed the ROC curves of original images, de-biased images and vesselness filtered images. As shown in Figure 7, optimal thresholding of the de-biased images can provide highly accurate segmentations, while slightly outperforming thresholding of the original images and more significantly of the vesselness filtered images. Regardless of the choice of the threshold, direct thresholding of vesselness filtered images can barely reach the sensitivity above 90%.

**FIGURE 8.**
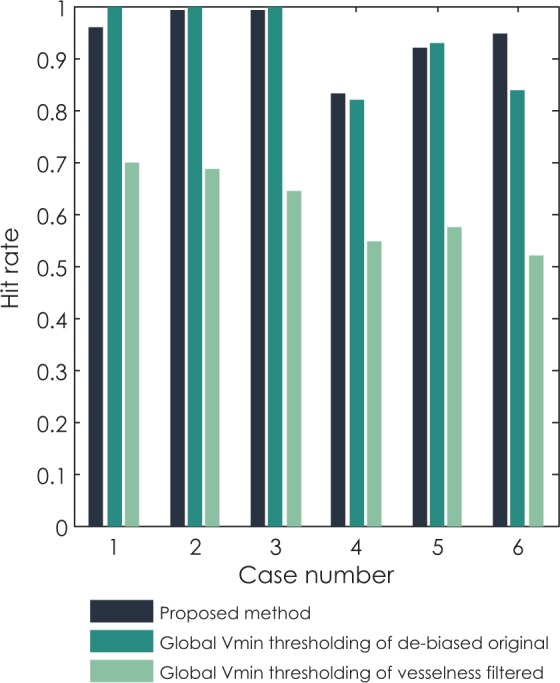
Comparison of hit rates for all six clinical cases segmented with three methods: the proposed method, global variance minimization thresholding of the original de-biased image and global variance minimization thresholding of the vesselness filtered image.

**FIGURE 9.**
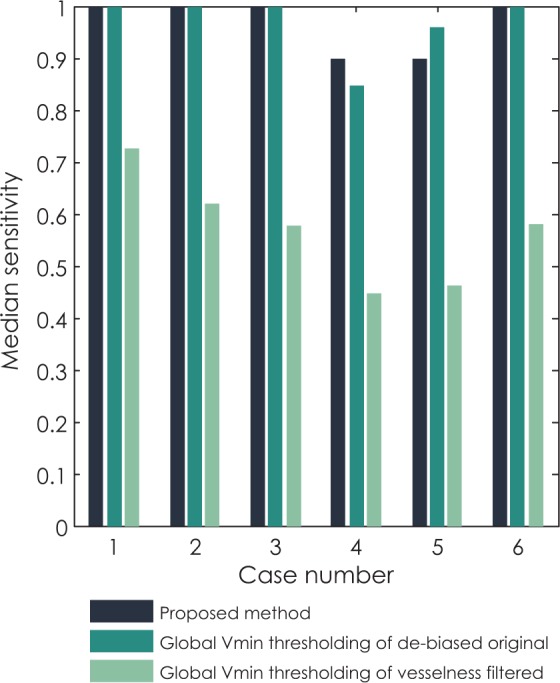
Comparison of median sensitivity for all six clinical cases segmented with three methods: the proposed method, global variance minimization thresholding of the original de-biased image and global variance minimization thresholding of the vesselness filtered image.

Final comparison of methods that performed best on phantoms (global thresholding of original de-biased images and vesselness filtered images based on variance minimization) and the proposed method based on local thresholding is given by observing hit rate, median sensitivity, median Hausdorff distance and median average symmetric surface distance (ASSD) per clinical case. In Figure 8 we can observe that methods based on global thresholding of the original de-biased images have a hit rate around or above 90%, while results of global thresholding of vesselness filtered images identify less than 70% of all objects. Similar results are observed for median sensitivity in Figure 9. The values of median sensitivity are again around or above 90% for global thresholding of original de-biased images and below 70% for global thresholding of vesselness filtered images.

**FIGURE 10.**
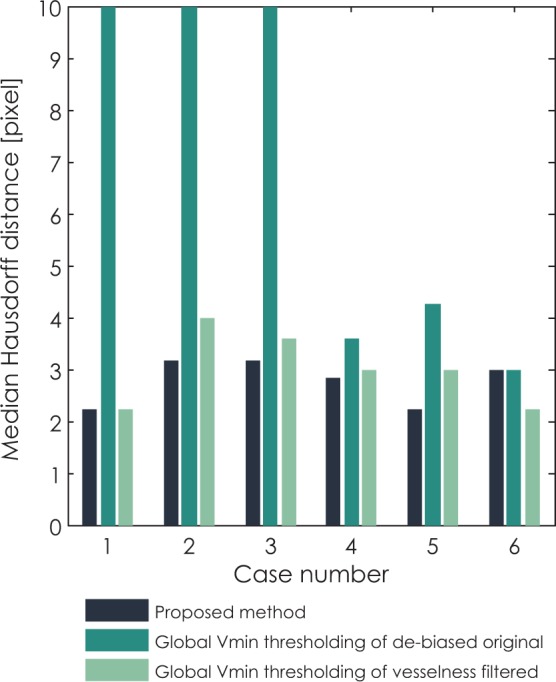
Comparison of median Hausdorff distance for all six clinical cases segmented with three methods: the proposed method, global variance minimization thresholding of the original de-biased image and global variance minimization thresholding of the vesselness filtered image.

**FIGURE 11.**
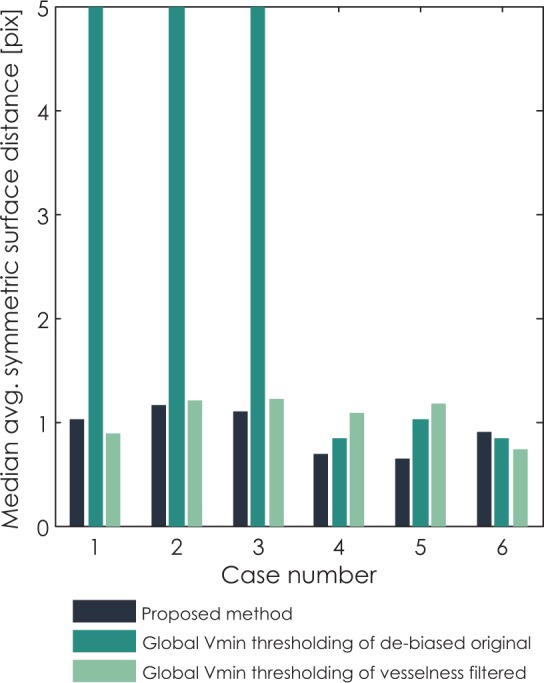
Comparison of median average symmetric surface distance (ASSD) for all six clinical cases segmented with three methods: the proposed method, global variance minimization thresholding of the original de-biased image and global variance minimization thresholding of the vesselness filtered image.

The differences between the proposed method and methods based on global thresholding can be observed through median Hausdorff distance and median ASSD in Figures 10 and 11, respectively. Values of median Hausdorff distance for the proposed method are for all cases in the range of around 2.2 pixels to 3.2 pixels. Values of median Hausdorff distance for global thresholding of original cases are in five out of six cases higher than those for the proposed method, in three cases even extremely high (above 10 pixel), indicating overestimation of the threshold on the global level. Values of median Hausdorff distance for thresholding of vesselness filtered images are mostly higher than those observed for the proposed method, ranging between 2.2 pixels and 4 pixels. As for median ASSD, values for all methods are in almost all cases below 1.2 pixels, except for an extreme for global thresholding in the same three cases which also provided extremes for the median Hausdorff distance.

Final quantitative results of our proposed segmentation method are given in Tables 2 and 3. In Table 2 we provide results of validating the segmentation of all vessels that are critical for electroporation-based treatments of liver metastases as indicated previously in the paper. The mean hit rate for these vessels is high: 96.7% of objects that build up main vessels were detected. The mean value of median sensitivity of the detected objects is 93.7%. The mean error measured through ASSD is 1 pixel, while maximum error in specificity expressed through mean Hausdorff distance reaches 4.26 pixels.

The statistics in Table 3 includes all the vessels that were marked by the radiologist. Median sensitivity nearly equals the one of main vessels with 96.65%. Mean value of median ASSD which equals 0.92 pixel and mean value of median Hausdorff distance which equals 2.77 pixels indicate smaller level of error in specificity for smaller, more regularly shaped vessels.

## Discussion

The main aim of the study described in this paper was to find a method which would successfully segment hepatic vessels from MRI images for the purpose of generating a patient-specific treatment plan for electroporation-based treatment like electrochemotherapy and non-thermal irreversible electroporation. The purpose for which the segmentation will be used has resulted in specific criteria the segmentation method must meet. Firstly, the segmentation method must detect all hepatic vessels that are considered critical in electroporation-based treatments of the liver, which are basically all major hepatic vessels with branches up to second order. Secondly, the method has to be robust. Thirdly, the method has to perform segmentation with minimal or no user interaction required. As for accuracy of the segmentation method, there were no limits posed prior to the beginning of the study. Having in mind that no segmentation method provides absolutely accurate results we rather aimed at finding the best method that would meet the set criteria and assess what is the maximum inaccuracy that method produces. Only after the maximum inaccuracy has been quantified we can conclude if segmenting hepatic vessels from MRI images for electroporation-based treatments is feasible or not.

The first step in finding a method that could segment hepatic vessels from MRI images while satisfying all the mentioned criteria was using the established methods already used on CT images. Since we intended to test several methods we needed means of evaluation that would enable objective comparison of segmentation results. We decided to start with a simple phantom model of a single vessel for detailed observation of methods’ behaviors and continue assessing robustness of methods on clinical data.

Our first choice of segmentation methods were the most widely used approaches of identifying vessels with vessel-enhancing filters and automatic thresholding. Regarding automatic thresholding, we have tested two different methods: thresholding based on intra-variance minimization and thresholding based on entropy maximization. Both thresholding methods were applied on both original de-biased and vesselness filtered images, thus resulting in in four different combinations. All four segmentation procedure combinations were run on both phantom data and clinical data. The results of evaluation on phantom data showed that intra-variance minimization thresholding applied on both original and vesselness filtered images of phantoms provides segmentation without errors. An entropy-maximizing thresholding applied on phantom data was not successful and showed tendency to over-estimate the optimal threshold, both for original and vesselness-filtered images.

Application of variance-minimization thresholding alone and in combination with vessel enhancement on clinical data did not provide satisfying results. Although variance-minimization thresholding on the level of the whole slice had a high hit-rate and sensitivity (as seen in Figures 8 and 9, respectively) it resulted in large oversegmentation error in half of clinical cases (seen in Figures 10 and 11). Namely, the large oversegmentation error appeared in clinical cases with larger image resolution. The reason for this is the fact that the difference between the size (expressed in number of pixels) of foreground (vessels) and the size of background (liver) is larger in images with higher resolution. Additionally, larger difference between foreground and background size also means larger difference between their variance, which was shown to cause oversegmentation of foreground objects when the intra-variance minimization thresholding method is applied.^65^–^67^ The other issue of variance-minimization thresholding applied on original images was that it detected not only vessels but also the liver border which had intensity values similar to vessels (Figure 6.C). Vessel enhancement on the other hand was not sensitive enough. The reason for such low sensitivity is primarily large slice thickness of the clinical data. With a gap of 3 mm between neighboring slices the changes in vessel forms are not smooth enough. Most cases where vessel enhancement was used so far had much smaller slice thickness, with sometimes even isotropic voxels.^20^,^30^,^32^,^33^,^36^ Also, hepatic vessels as seen in medical images, especially major vessels, do not have a shape of a perfect tube. These shape irregularities result in smaller values of vesselness for large vessels which can then not be detected by automatic thresholding.

The complementing weaknesses of vessel enhancement and thresholding on a global level resulted in a segmentation method that would combine these two approaches in a different way. The proposed segmentation method utilizes vessel enhancement thresholded with a low threshold to determine the existence of a vessel. The automatic thresholding method then performs the thresholding in a small region of interest just around the location of a vessel detected in the first step. Regarding the dimension in which each step of the proposed method was performed, we have utilized 3D information only to determine the basic vessel model. For determining the accurate vessel borders we relied on the original 2D information in order to avoid interpolation necessary to obtain isotropic voxels.

The evaluation of the proposed method on clinical data resulted in no large over-segmentations (Figures 10 and 11) with high values of hit-rate (Figure 8) and sensitivity (Figure 9) for both major vessels and all vessels together (tables 2 and 3, respectively). Value of average symmetric surface distance indicates that an error in segmentation of any vessel, major or small is mostly likely to be smaller than 1 pixel. Value of the median Hausdorff distance indicates that, if a larger segmentation error, i.e. an *outlier* appears, it is most likely to be 4.3 pixel for major vessels and 2.8 pixel in general, as seen in Tables 2 and 3, respectively.

A comparison with results of previously developed methods for segmentation of hepatic vessels from CT images was difficult. The general obstacle for such comparison is lack of a publicly available database that all methods would be tested on as well as the lack of unified, standard criteria for validation. Some attempts to standardize validation that would enable direct comparison were made through MICCAI grand challenges which were already organized for segmentation of liver and liver tumors from CT images.^61^,^68^ A similar grand challenge yet remains to be organized for segmentation of hepatic vessels from images of any modality. The main obstacle to performing any kind of comparison of our results was the fact that there are not many results to be compared with. We have searched the Web of Science directory for all papers on the topic of hepatic/liver vessel segmentation from CT images, along with referenced and referencing papers of matches. The search yielded only 19 matches in the past 20 years. Out of 19 papers, 17 of them were tested on clinical data while the remaining 2 were tested only on phantom data. Out of the mentioned 17 papers only 1 paper^39^ included a detailed evaluation similar to ours in which the authors assessed the method accuracy and error in the form of average distance as we have. The results from that paper report an average distance of 0.9 mm to 4.4 mm, which is comparable to our findings. The authors also state that »the misclassified vessels do not deviate from the ground truth far away«.^39^ In the majority of the remaining 16 papers the evaluation was qualitative based on visual inspection of “goodness” of the segmentation.

Although no definite conclusions can be made about the validity of our method based on direct comparison with other methods, positive conclusions can be drawn with respect to the criteria set at the beginning of our research. Firstly, the method we propose is able to detect all vessels critical for electroporation-based treatments of liver with above 93% sensitivity. The errors that are produced in segmentation of critical vessels appear in amount which is sufficiently low to enable a fast correction by an expert radiologist. Namely, manual validation by an expert radiologist is a step that should still be mandatory in the process of treatment planning and would also provide valuable feedback for improvement of the segmentation method.

Secondly, the proposed segmentation method is robust to variations in image resolution, imaging devices and protocols. We have shown that the method consistently provided results of high sensitivity when validated on images of different intra-slice resolution. Regarding the slice thickness the results could only be improved using smaller thickness while we would not suggest using images with slices thicker than 3 mm because of high complexity of hepatic vasculature. Given the fact that main method parameters (namely thresholds and parameter *c* that regulates response of the vesselness filter to high contrast) are automatically calculated from the image, the method is expected to perform well regardless of the imaging device. In order to be used on image series imaged with different protocols only one change should be made. Should the vessels in such case appear brighter instead of darker against the background a minus sign should be added in front of the equation for the vesselness filter.

Thirdly, the method is performed completely automatically with no user input, assuming that the liver is segmented automatically.^17^

Finally, the evaluation of the proposed method resulted in a quantitative estimation of a segmentation error which is most likely to appear in the worst case of segmentation – 2.8 pixels. Assuming the lowest image resolution of our test images, which was 1.188 mm per pixel, the above mentioned segmentation error would translate to 3.3 mm. For comparison, a study on registering CT with MRI images of the liver performed in 2005 reports a mean registration error of 14.0–18.9 mm,^69^ while a newer study from 2010 reports a mean error of 3.3 mm.^70^ If a segmentation of hepatic vessels was performed on CT images and then registered with MRI images for colorectal metastases segmentation the total error of vessel model would be even higher due to the error of segmentation on CT images itself. It is thus indeed more feasible to perform the hepatic vessel segmentation with our proposed method directly on MRI images. Still, in order to give a final decision if the proposed method of segmentation of hepatic vessels from MRI images can be used in treatment planning of liver tumors with electroporation-based treatments an additional assessment is needed. The additional assessment would be based on introducing the estimated value of the segmentation error – 2.8 pixels – into treatment plan calculations and observe its influence on the distribution of the electric field.

## Figures and Tables

**TABLE 1.** Sequential list of all the steps performed within the proposed optimal method, along with inputs, outputs and parameters of each step and the dimension (2D or 3D) in which the step is performed. *(Ox)* denotes an output from a previous step where x is the step number

**No**	**Step**	**Input**	**Output**	**Parameters**	**Dimension**
1	Bias removal	Original unmasked image	De-biased image (O1)	/	2D
2	Sinc interpolation to obtain isotropic voxels	De-biased image (O1) Liver mask	Interpolated de-biased image (O2’) Interpolated liver mask (O2’’)	/	3D
3	Masking	Interpolated de-biased image (O2’) Interpolated liver mask (O2’’)	Interpolated masked de-biased image (O3)	/	2D
4	Frangi filtering	Interpolated masked de-biased image (O3)	Interpolated vesselness filtered image (O4)	Gaussian kernel σ=[1,12] with a step of 0.5 α=0.3 β=0.7 c= half of Frobenius norm	3D
5	Interpolation to original voxel size	Interpolated vesselness filtered image (O4)	Vesselness filtered image (O5)	/	3D
6	Masking	Vesselness filtered image (O5) Liver mask	Masked vesselness filtered image (O6)	/	2D
7	Thresholding with a low threshold	Masked vesselness filtered image (O6)	Basic vessel model (O7)	Threshold = 0.05 * max(vesselness)	3D
8	Removal of small objects	Basic vessel model (O7)	Basic vessel model with objects with diameter > 3 mm (O8)	Size of small object = number of pixel of a circle with 3 mm diameter	2D
9	Connected component analysis	Basic vessel model with objects with diameter > 3 mm (O8)	Basic objects (O9)	/	2D
10	Dilation	Basic object (O9)	ROI of object (O10)	Structuring element: disc with radius = 5	2D, per object
11	Masking	De-biased image (O1) Liver mask	Masked de-biased image (O11)	/	2D
12	Local thresholding	ROI of object (O10) Masked de-biased image (O11)	Locally thresholded image (O12)	Threshold determined for each ROI through variance minimization	2D, per object
13	Region growing	Locally thresholded image (O12) Masked de-biased image (O11)	Region grown image (O13)	Threshold = median of locally thresholded image, per slice 27-neighborhood	2D/3D
14	Erosion	Liver mask	Eroded mask (O14)	Structuring element: disc with radius = 6	2D
15	Masking	Region grown image (O13) Eroded mask (O14)	Segmented image (O15)	/	2D
16	Removal of small objects	Segmented image (O15)	Segmented image with objects with diameter > 3 mm (O16)	Size of small object = number of pixel of a circle with 3 mm diameter	2D

**TABLE 2.** Results of segmentation of major hepatic vessels only from six clinical cases. Segmentation was performed by the method based on local thresholding. Results show hit rate of all objects in all slices, median sensitivity (SEN), median average symmetric surface distance and median Hausdorff distance

**CASE**	**Number of objects**	**Pixel resolution [mm]**	**Hit rate [%]**	**Median SEN**	**Median ASSD [pix]**	**Median ASSD [mm]**	**Median Hausdorff distance [pix]**	**Median Hausdorff distance [mm]**
1	43	0.684	92.9	98.0	1.3	0.9	4.4	3.0
2	69	0.684	98.4	96.4	1.1	0.7	3.6	2.5
3	38	0.684	100.0	100.0	1.1	0.7	4.1	2.8
4	31	1.188	96.4	85.3	0.7	0.8	2.2	2.7
5	31	1.188	92.3	84.2	1.3	1.5	8.1	9.6
6	25	1.188	100.0	98.2	0.6	0.7	3.2	3.8
**OVERALL (mean)**			**96.7**	**93.7**	**1.0**	**0.9**	**4.3**	**4.1**

**TABLE 3.** Results of segmentation of all hepatic vessels from six clinical cases. Segmentation was performed by the method based on local thresholding. Results show median sensitivity (SEN), median average symmetric surface distance and median Hausdorff distance

**CASE**	**Number of objects**	**Pixel resolution [mm]**	**Median SEN**	**Median ASSD [pix]**	**Median ASSD [mm]**	**Median Hausdorff distance [pix]**	**Median Hausdorff distance [mm]**
1	305	0.684	100.0	1.0	0.7	2.2	1.5
2	347	0.684	100.0	1.2	0.8	3.2	2.2
3	328	0.684	100.0	1.1	0.8	3.2	2.2
4	327	1.188	89.9	0.7	0.8	2.8	3.4
5	400	1.188	90.0	0.6	0.8	2.2	2.7
6	454	1.188	100.0	0.9	1.1	3.0	3.6
**OVERALL (mean)**			**96.7**	**0.9**	**0.8**	**2.8**	**2.6**
